# Illness perceptions, experiences of stigma and engagement in functional neurological disorder (FND): exploring the role of multidisciplinary group education sessions

**DOI:** 10.1136/bmjno-2024-000633

**Published:** 2024-06-05

**Authors:** Cate Bailey, Niruj Agrawal, Sarah Cope, Barnaby Proctor, Bridget Mildon, Matt Butler, Kate Holt, Mark Edwards, Norman Poole, Timothy R Nicholson

**Affiliations:** 1 Neuropsychiatry Research and Education Group, Institute of Psychiatry Psychology & Neuroscience, King's College London, London, UK; 2 East London NHS Foundation Trust, London, UK; 3 Neuropsychiatry Service, South West London and St George's Mental Health NHS Trust, London, UK; 4 Atkinson Morley Regional Neurosciences Centre, St George's University Hospitals NHS Foundation Trust, London, UK; 5 FND Hope International, Salmon, Idaho, USA; 6 Wolfson Neurorehabilitation Centre, Queen Mary's Hospital, London, UK; 7 The Lishman Unit (Brain Injury and Functional Neurology), South London and Maudsley NHS Foundation Trust, London, UK

**Keywords:** functional neurological disorder, clinical neurology

## Abstract

**Background:**

A critical first step in managing functional neurological disorder (FND) is a positive diagnosis and clear explanation using an understandable illness model. Multidisciplinary group education sessions are one way to achieve this, with some evidence they improve understanding, confidence in diagnosis and outcomes with further treatment. In many conditions, illness perceptions and stigma affect distress, functioning, quality of life and engagement. Exploring relationships between these factors could lead to deeper understanding of the impact of education.

**Methods:**

Questionnaires assessing illness perceptions, quality of life, mood, anxiety, comorbidities, treatment engagement and stigma (both experienced and anticipated) were completed before, immediately and 1 month after a multidisciplinary online group education session for FND at a regional neurosciences centre. Free-text data on causal attributions and needs were also collected.

**Results:**

166 patients attended online education sessions from January 2022 to July 2023; 61 (37%) completed presession surveys, 42 (25%) completed postsession and 35 (21%) completed 1 month postsession surveys. Patients reported multiple comorbidities, poor quality of life, functioning and high levels of stigma. Illness perception scores indicated FND as threatening, mysterious and unpredictable, with low personal or treatment control over symptoms. Illness coherence/understanding (mean difference 2.27, p<0.01, 95% CI 1.22 to 4.23) and engagement (mean difference 2.42, p<0.01, 95% CI 0.46 to 4.36) increased after the session. There were no significant changes in stigma, distress, sense of control or anticipated discrimination. Free-text analysis revealed stress and trauma as the most common causal attributions, followed by physical illnesses. Patients requested personalised formulations, practical disability advice, help with explaining the condition to others (eg, employers), peer support and treatment.

**Conclusion:**

Multidisciplinary group FND education sessions potentially improve patient understanding and engagement. Clinicians should consider the possible benefits of personalised formulations and linking to practical and peer support. Further work assessing illness perceptions is needed, such as adapting measures for FND.

**WHAT IS ALREADY KNOWN ON THIS TOPIC**

- People with functional neurological disorder (FND) report limited understanding of their condition, poor quality of life and high levels of stigma but there has been little research exploring these issues, including how they might be improved by multidisciplinary education sessions.

**WHAT THIS STUDY ADDS**

- We provide preliminary evidence for illness perceptions being in dynamic relation with experiences and expectations, including stigma and discrimination within health, family and work environments.
- Multidisciplinary education may increase illness understanding and engagement, however people with FND also need personalised formulation and treatment, access to peer and practical support.

**HOW THIS STUDY MIGHT AFFECT RESEARCH, PRACTICE OR POLICY**

- Neuropsychiatry services should explore options for developing multidisciplinary education sessions, which offer increased personalisation, links to practical disability advice and peer support.
- Interventions which address stigma and discrimination towards people with FND in health systems and work environments should be a focus of future research.
- Adapted tools for measuring illness perceptions in FND should be developed to improve nuance and acceptability.

## Background

Functional neurological disorder (FND) is defined by neurological symptoms (eg, paralysis, seizures, movement disorders, sensory loss) where history and examination indicate the disorder is of nervous system functioning rather than structural damage.^1^ Although FND accounts for between 6% and 16% of neurology outpatient consultations,^2 3^ patients often experience long delays in diagnosis and treatment,^4^ significant disability and poor quality of life.^5^

As in any condition, explaining the diagnosis is a critical first step in management^6–8^ and facilitates treatment engagement and self-help.^7^ Informed by neuroscientific and clinical evidence nuanced bio-psycho-social FND models have replaced simplistic psychological ‘conversion’ explanations.^9 10^ These newer models give insight into how triggers such as initial illness, injury, stress and/or trauma may lead to abnormal sensorimotor processing, body-focussed attention and impaired sense of agency.^9 10^ Importantly, illness beliefs and expectations likely contribute to how symptoms develop or are maintained.^9 11^

Illness perceptions describe a person’s beliefs and expectations about their condition and are linked to coping, engagement and health outcomes in FND^11^ and a range of other conditions.^12^ People with functional seizures and weakness report low levels of personal control and symptom understanding.^13^ A recent systematic review found threatening illness perceptions in both functional and epileptic seizures were associated with poorer clinical outcomes and quality of life.^11^

FND is a challenging diagnosis to communicate because of aetiological complexity and limited awareness in clinicians and wider society.^14^ Despite neuroscientific advances in understanding mechanisms, clinicians describe uncertainty and sometimes stigmatising attitudes^15^ stemming in part from limited undergraduate and postgraduate education.^16^ This contributes to difficult consultations where patients may be disbelieved and dismissed by clinicians.^17^

Limited and variable FND services, being a ‘hidden’^18^ and stigmatised condition,^19^ suggests that group education sessions could provide opportunities to improve understanding, illness perceptions and treatment engagement.^14^ Education sessions providing accurate information about illnesses and treatment have been linked to improvements in self-efficacy, self-management and carer/family understanding.^20^

A multidisciplinary (MDT), cognitive–behavioural therapy (CBT)-informed FND group education session, using a bio-psycho-social explanatory model, has been conducted by the neuropsychiatry service at St. George’s Hospital, London since 2016.^21^ Previous data from 193 patients and 153 relatives found high satisfaction and significant increases in understanding, acceptance, hopefulness and belief in treatability of FND after the session.^21^ However, the study used single question Likert measures and identified a need for further evaluation using validated measures of illness perceptions and longer follow-up.^21^

FND group education sessions could be a cost-effective addition to FND pathways but their impact on outcomes and illness perceptions has not been studied using validated measures of illness perceptions or engagement, nor considered whether effects are sustained. We sought to examine illness perceptions in FND group education session attendees and relationships with comorbidities, quality of life and experiences of stigma. A secondary aim was to consider how education might influence illness perceptions and engagement.

## Methods

### Education session

Full details of the session are described previously.^14 21^ The 1 hour and 45 min session includes presentations from four MDT members (neurologist, neuropsychiatrist, psychologist, neurophysiotherapist) and an expert by experience. Sessions attract approximately 50 attendees with FND (plus carer/family members) every 3 months. Presenters cover assessment, diagnosis, common symptoms, aetiology and treatments. Two fictional FND subtype scenarios illustrate bio-psycho-social CBT formulations. There are opportunities to ask questions, and summary handouts containing links to online educational, self-help and peer support resources are provided. Sessions were previously run face-to-face, but moved online during the COVID-19 pandemic.

### Recruitment and patient input

All patients referred to the St George’s Hospital Neuropsychiatry service with a diagnosis of FND made by a neuropsychiatrist or neurologist were eligible if they were >18 years of age, able to consent and fluent in English. Invitees received written and video study information. Interested participants completed online consent forms and baseline surveys before the session. Participants were sent postsession surveys immediately after the session and 1 month later. Only participants who completed baseline surveys were sent postsession surveys. A single prompt was sent to non-responders. Participants who completed any survey part were sent a £10 thank you voucher.

This study was developed with input from patient charity (FND Hope) scientific committee who helped design sensitive questions about ‘beliefs’ and ‘illness perceptions’ in the context of many individuals having their experiences dismissed or disbelieved.

### Questionnaires

Data were collected using the Qualtrics online platform (see online supplemental appendix 1 for full questionnaire).

10.1136/bmjno-2024-000633.supp1 Supplementary data

#### Self-reported symptoms

Primary FND and other symptoms were listed based on a recent large online FND survey.^22^ An international consensus ‘core outcome measure set’^23^ informed instrument choice.

#### Illness perceptions

*Revised Illness Perceptions Questionnaire (IPQ-R)*: an 84-item measure based on Leventhal’s ‘common sense model of self-regulation’.^24^ Its use in chronic pain, neurological and mental health conditions has shown it is sensitive to change.^24^ Sections cover ‘identity’ (illness-associated symptoms) and ‘causes’ (18 listed standardised causes). A free-text box invites participants to describe the most important causes of their illness. Subscales address theoretical components of illness representations including: (1) consequences (the impact of the illness), (2) timeline (acute/chronic) (the perception of prognosis), (3) timeline (cyclical) (perception of the cyclical nature of illness), (4) personal control (perceptions of ability to control the illness), (5) treatment control/cure (perceptions of treatment efficacy), (6) illness coherence (perceptions of illness understanding) and (7) emotional representations (perceptions of the effect of illness on mood). Researchers are encouraged to adapt questionnaires; replacing ‘illness’ with the relevant condition.^24 25^ High scores on identity, consequences, timeline acute/chronic and cyclical subscales represent strong beliefs about number of symptoms attributed, negative consequences, chronicity and unpredictability of the condition.^24^ High scores on control and coherence subscales represent positive beliefs about controllability and personal understanding.^24^

#### Stigma

*Scale for Stigma in Chronic Illness (SSCI-8)*: measures both enacted and internalised stigma.^26^ Enacted stigma describes negative attitudes expressed by members of the public (including healthcare professionals) with higher scores linked to reduced quality of life in some neurological conditions.^26^ Internalised stigma occurs when an individual perceives enacted stigma and incorporates negative evaluations into the self.^26^ It is related to poor health outcomes, quality of life, low self-esteem and self-efficacy.^26^

*Chronic Illness Anticipated Stigma Scale (CIASS):* Measures anticipated stigma; a person’s expectations of prejudice, stereotyping and discrimination.^27^ CIASS asks about future interactions with friends, family, employers and healthcare professionals.^27^

#### Other measures

*Patient Health Engagement (PHE) scale*: assesses engagement and is validated in fibromyalgia and rheumatoid arthritis.^28^ Improving patient understanding of their condition and empowering self-management is considered a factor in outcomes and satisfaction with care.^28^

*Short Form 36 (SF-36):* assesses disability via eight health concepts exploring limitations in functioning, role, sense of vitality and pain.^29^ Higher scores indicate greater health-related quality of life and functioning.

*Hospital Anxiety and Depression Scale (HADS):* depression and anxiety screen validated in clinical populations^30^ including FND.^31^

All measures were collected at baseline but only IPQ-R, SSCI-8 and CIASS repeated immediately postsession and at 1 month. A free-text box asked participants what questions they had about their FND at the three time points and what help or support they needed.

### Analyses

Statistical analyses were performed with SPSS Statistics, V.29.0.1.0 (IBM). Missing data were assessed using Little’s Missing Completely At Random (MCAR) test and independent sample t-tests compared baseline characteristics and measures for participants who did or did not complete follow-up. Spearman’s correlation coefficients investigated relationships between illness perceptions and mood, quality of life and stigma. Repeated-measure analysis of variance with Bonferroni corrections assessed differences between measures for participants who provided data for each timepoint: T0 (pre-education session), T1 (immediately post-education session) and T2 (1 month after the education session).

A content analysis^32^ using NVIVO (V.12) was performed for qualitative data including IPQ-R responses, free-text questions and needs. CB coded all data line-by-line before categorising in Excel. CB and NP reviewed data and agreed overall categories across all time points noting changes in requests for information or support. Free-text causes were grouped by content and frequency, and changes in individual causal attributions across timepoints.

## Results

166 people attended online education sessions from January 2022 to July 2023; 61 (37%) completed baseline surveys, 42 (25%) completed immediate and 35 (21%) (54% of baseline cohort) completed 1 month follow-up. Little’s MCAR indicated missing data was randomly distributed and there were no statistically significant differences between those who completed baseline and follow-up surveys and those who did not, on gender, comorbidities, disability, mood, stigma and illness perceptions.

### Sociodemographic and clinical characteristics

Sociodemographic characteristics are shown in table 1 and FND symptoms and duration in table 2.

**Table 1:** Demographic information

	N	%
Gender		
Man	16	26.2
Woman	44	72.1
Trans-woman	1	1.6
Age (years)		
18–25	14	23.0
26–35	12	19.7
36–45	10	16.4
46–55	14	23.0
56–65	7	11.5
66+	4	6.6
Ethnicity		
Asian Pakistani	1	1.6
Asian Bangladeshi	1	1.6
White British	42	68.9
Any other white background	6	9.8
Black African	4	6.6
Black Caribbean	3	4.9
White and black Caribbean	4	6.6
Education level		
Primary school	2	3.3
GCSEs or equivalent	12	19.7
A-levels or equivalent	19	31.1
University undergraduate degree	14	23.0
University postgraduate degree	13	21.3
Missing	1	1.6
Current employment or leave		
Full time work	13	21.3
Part time work	11	18.0
Retired (due to age)	4	6.6
Retired (due to medical condition)	10	16.4
Volunteer work	1	1.6
Long-term sick leave (>3 months)	15	24.6
Student	6	9.8
Missing	1	1.6

**Table 2:** Primary FND symptom and duration

	N	%
Primary FND symptom		
Movement disorder	8	13.1
Gait disturbance	8	13.1
Tremor	3	4.9
Weakness or paralysis	9	14.8
Muscle dystonia	5	8.2
Seizures or fits	15	24.6
Memory or thinking problems	5	8.2
Numbness or tingling	3	4.9
Other (dissociation, fatigue, speech)	5	8.2
Duration of symptoms		
3–6 months	3	4.9
6–12 months	14	23.0
1–5 years	27	44.3
>5 years	17	27.8

FND, functional neurological disorder.

**Figure 1:**
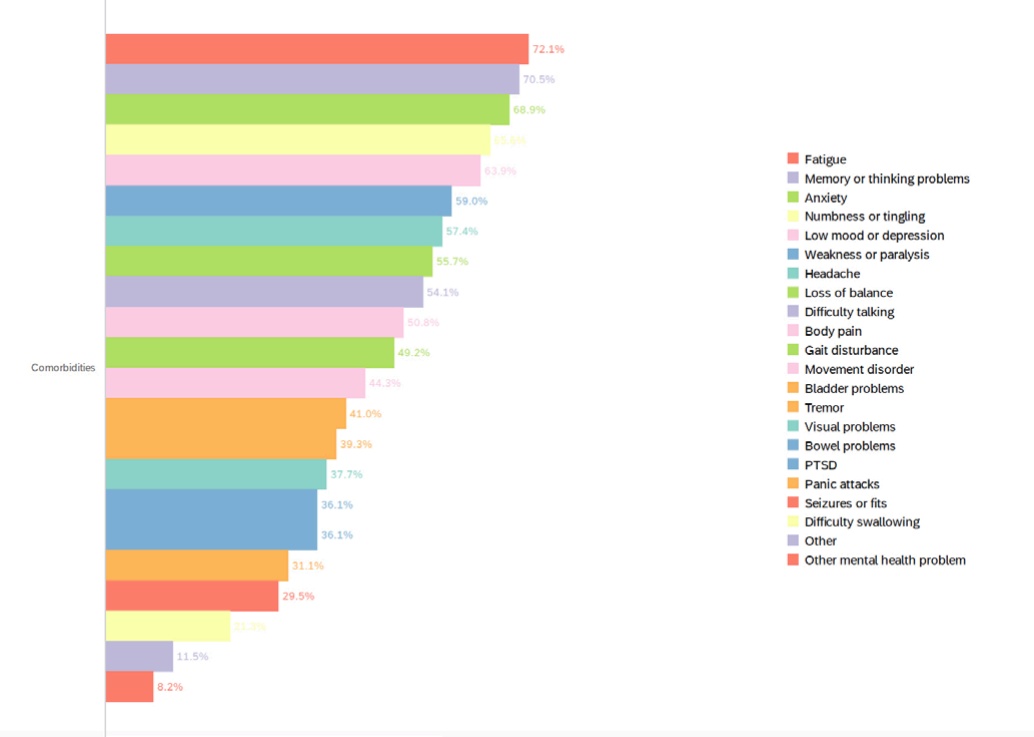
Comorbidities reported by participants at baseline (n= 61). PTSD, post-traumatic stress disorder.

The majority of participants were women (72.1%), white British (68.9%) and not in full time work (78.3%), with 24.6% on long-term sick leave. Seizures (24.6%) were the most common primary FND symptom identified, with other subtypes broadly represented. The majority of participants (95.1%) reported having FND for at least 6 months, 44.3% for 1–5 years and 27.9% for >5 years. Fatigue was the most common comorbid symptom (72.1%), followed by memory or thinking problems (70.5%) and anxiety (68.9%) (figure 1). 52.5% of participants had anxiety scores meeting the abnormal threshold HADS, while 35.6% met caseness for depression (table 3). Mean scores for SF-36 were below average for most areas, except for average scores on emotional well-being (50.0%). Physical role limitation (20.5%), fatigue (26.0%), emotional role limitation (36.6%) were most affected.

**Table 3:** SF-36 and HADS scores

SF-36	N	Mean	SD
Physical functioning	60	48.8	33.3
Physical role limitation	61	20.5	33.1
Emotional role limitation	61	36.6	44.2
Fatigue/Energy	56	26.0	20.5
Emotional well-being	61	50.0	20.6
Social functioning	61	38.7	28.8
Pain	61	43.6	34.4
General health	60	39.7	27.7

Hospital anxiety and depression	N	Mean	SD	Normal %	Borderline %	Case %
Anxiety subscore	59	10.7	5.5	32.2	15.3	52.5
Depression subscore	59	8.5	4.6	40.7	23.7	35.6
HADS scores: normal 0–7, borderline 8–10, abnormal/case 11–21						

HADS, Hospital Anxiety and Depression Scale; SF-36, Short Form 36.

### Stigma

Enacted and internalised stigma, measured with SSCI-8 is shown in table 4 and online supplemental appendix 2.

10.1136/bmjno-2024-000633.supp2 Supplementary data

**Table 4:** SSCI-8 and CIASS scores

	Mean	SD	95% CI		
SSCI-8 (out of 40)					
Baseline	20.7	1.1	18.5	23.0	Maximum possible score=40
Immediately after session	22.3	1.2	19.9	24.7	Minimum possible score=8
1 month after session	20.8	1.2	18.4	23.2	
CIASS—healthcare professionals (out of 20)					
Baseline	11.5	0.9	9.7	13.3	
Immediately after session	11.8	0.9	9.9	13.6	
1 month after session	12.0	0.9	10.2	13.8	
CIASS—friends and family (out of 20)					
Baseline	8.7	0.9	6.9	10.5	
Immediately after session	9.9	0.9	7.9	11.8	
1 month after session	9.2	0.9	7.4	11.0	
CIASS—employers and colleagues (out of 20)					
Baseline	12.4	0.8	10.7	14.1	Maximum possible score=20
Immediately after session	12.9	0.9	11.0	14.5	Minimum possible score=4
1 month after session	14.0	0.7	12.5	15.4	

N completing presession survey=59.

N completing postsession survey=43.

N completing 1 month postsession survey=42.

N completing survey at three timepoints=34.

CIASS, Chronic Illness Anticipated Stigma Scale; SSCI-8, Scale for Stigma in Chronic Illness.

Scores were high, with no significant gender difference and no change across time points. Almost half participants (46.7%) reported feeling embarrassed about FND and while only 10.2% reported feeling people were often or always unkind to them because of FND. Measured using CIASS, participants anticipated more stigma and discrimination from healthcare professionals and employers than from their families. 23% of participants thought it was ‘somewhat’ to ‘very likely’ that a friend or family member would be angry with them because of their FND. In contrast, 63.3% thought it ‘somewhat’ to ‘very likely’ that a healthcare professional would be frustrated with them. 59.3% thought it was ‘somewhat likely’ to ‘very likely’ that a healthcare professional would give them poor care. 59.3% of participants thought it was ‘somewhat’ to ‘very likely’ a colleague would discriminate against them. 62.71% felt it was ‘somewhat’ to ‘very likely’ they would not be promoted. There were no significant changes in anticipated stigma after the education session. CIASS frequencies are found in online supplemental appendix 3.

10.1136/bmjno-2024-000633.supp3 Supplementary data

### Engagement

PHE scores are shown in table 5.

**Table 5:** IPQ-R subscales and PHE at three time points

	Mean	SD	95% CI		Range of possible scores
			Lower	Upper	
IPQ-R identity subscale					
Baseline	6.64	0.49	5.64	7.65	0–14
Immediately after	6.18	0.58	4.99	7.37	
1 month after	6.64	0.49	5.64	7.65	
IPQ-R timeline acute-chronic					
Baseline	22.52	0.70	21.10	23.93	6–30
Immediately after	**21.58**	0.77	20.01	23.14	
1 month after	23.21*	0.80	21.58	24.85	
IPQ-R timeline cyclical					
Baseline	13.85	0.69	12.45	15.25	4–20
Immediately after	13.76	0.61	12.51	15.01	
1 month after	13.58	0.68	12.19	14.96	
IPQ-R consequences					
Baseline	23.88	0.65	22.57	25.19	6–30
Immediately after	23.97	0.74	22.47	25.47	
1 month after	23.18	0.80	21.55	24.81	
IPQ-R personal control					
Baseline	20.03	0.83	18.34	21.72	6–30
Immediately after	21.15	0.76	19.60	22.71	
1 month after	20.33	0.79	18.73	21.94	
IPQ-R treatment control					
Baseline	15.52	0.56	14.37	16.66	5–25
Immediately after	15.73	0.59	14.52	16.93	
1 month after	15.64	0.67	14.28	17.00	
IPQ-R emotional representation					
Baseline	22.15	0.97	20.17	24.14	6–30
Immediately after	20.79	0.94	18.88	22.70	
1 month after	20.46	1.00	18.41	22.50	
IPQ-R illness coherence					
Baseline	**13.03**	0.71	11.59	14.48	5–25
Immediately after	15.76*	0.82	14.09	17.43	
1 month after	15.52*	0.82	13.85	17.18	
PHE					
Baseline	**17.21**	1.09	14.99	19.42	5–35
Immediately after	19.62*	0.91	17.76	21.47	
1 month after	19.27	1.02	17.18	21.35	

N completing presession survey=60.

N completing immediate postsession survey=43.

N completing 1 month postsession survey=41.

N completing all measures at three time points=33.

*Significant change.

IPQ-R, Illness Perceptions Questionnaire Revised; PHE, Patient Health Engagement.

Engagement scores were low at baseline (mean 17.2 out of possible 35) and at follow-up (mean 19.6 immediately following the session and 19.3 1 month later). When asked to think about their FND, 52.6% of participants endorsed responses ranging from “I feel anxious every time a new symptom arises” to “I feel overwhelmed by emotions”. There was a small, statistically significant increase in engagement after the education session (14% mean change p<0.05), which was no longer significant at 1 month (12% mean change p=0.07) (table 5).

### Illness perceptions

IPQ-R subscales are shown in table 5. FND was perceived as distressing and threatening; for example, on individual questions 58.3% agreed/strongly agreed that FND made them feel afraid and 51.7% agreed/strongly agreed FND had serious financial consequences. 85% agreed/strongly agreed it had major life consequences. High scores (22.5 mean score out of a possible 30) on the IPQ-R acute/chronic timeline subscale suggested participants felt FND would last a long time with 51.7% expecting it to be lifelong. FND was also perceived as unpredictable as measured on the timeline cyclical subscale (13.8 mean score out of a possible 20).

Illness coherence was also low at baseline (mean score 13.0 out of possible 25) with only 20% of participants agreeing/strongly agreeing to a clear understanding of their FND. 78.8% strongly agreed/agreed their FND was puzzling. There was a statistically significant increase in illness coherence (2.72 mean difference, 20.9% mean change, p<0.01) preserved at 1 month (2.25 mean difference 16.9% mean change, p<0.01) after the education session. The only other statistically significant change was a small increase in sense of FND lasting a long time (timeline—acute/chronic); between immediate follow-up and 1 month (mean change 1.64, 7.25% change p<0.04). Relevant change calculations are shown in table 6.

**Table 6:** Mean IPQ-R and PHE scores at baseline, immediately after the session and 1 month postsession, adjusted for repeated measures using Bonferroni correction

	Mean difference from baseline	Mean % Δ from baseline	SE	Sig.b	95% CI difference	
Baseline IPQ-R acute-chronic compared with						
Immediate	−0.94	Not significant	0.70	0.568	−2.71	0.83
1 month	0.70	Not significant	0.70	0.977	−1.07	2.46
Immediate IPQ-R acute-chronic compared with						
1 month	1.64*	7.25	0.47	0.004	0.45	2.82
Baseline IPQ-R coherence compared with						
Immediate	2.73*	20.92	0.60	<0.001	1.22	4.23
1 month	2.25*	16.90	0.57	<0.001	1.05	3.92
Baseline PHE coherence compared with						
Immediate	2.41*	14.01	0.77	0.01	0.46	4.36
1 month	2.06	11.97	0.87	0.07	0.15	4.26

*For significant change.

IPQ-R, Illness Perceptions Questionnaire Revised; PHE, Patient Health Engagement.

### Causes of FND

In the IPQ-R, participants rate their agreement with a standard list of illness causes. Frequencies of endorsement for IPQ-R causes are provided in online supplemental appendix 4. The most highly endorsed IPQ-R cause was ‘stress or worry’ which 70.0% agreed or strongly agreed with. 51.7% agreed/strongly agreed their emotional state caused their FND, 45.0% agreed or strongly agreed that family problems caused their FND. 31.7% agreed/strongly agreed poor medical care was the cause.

10.1136/bmjno-2024-000633.supp4 Supplementary data

**Figure 2:**
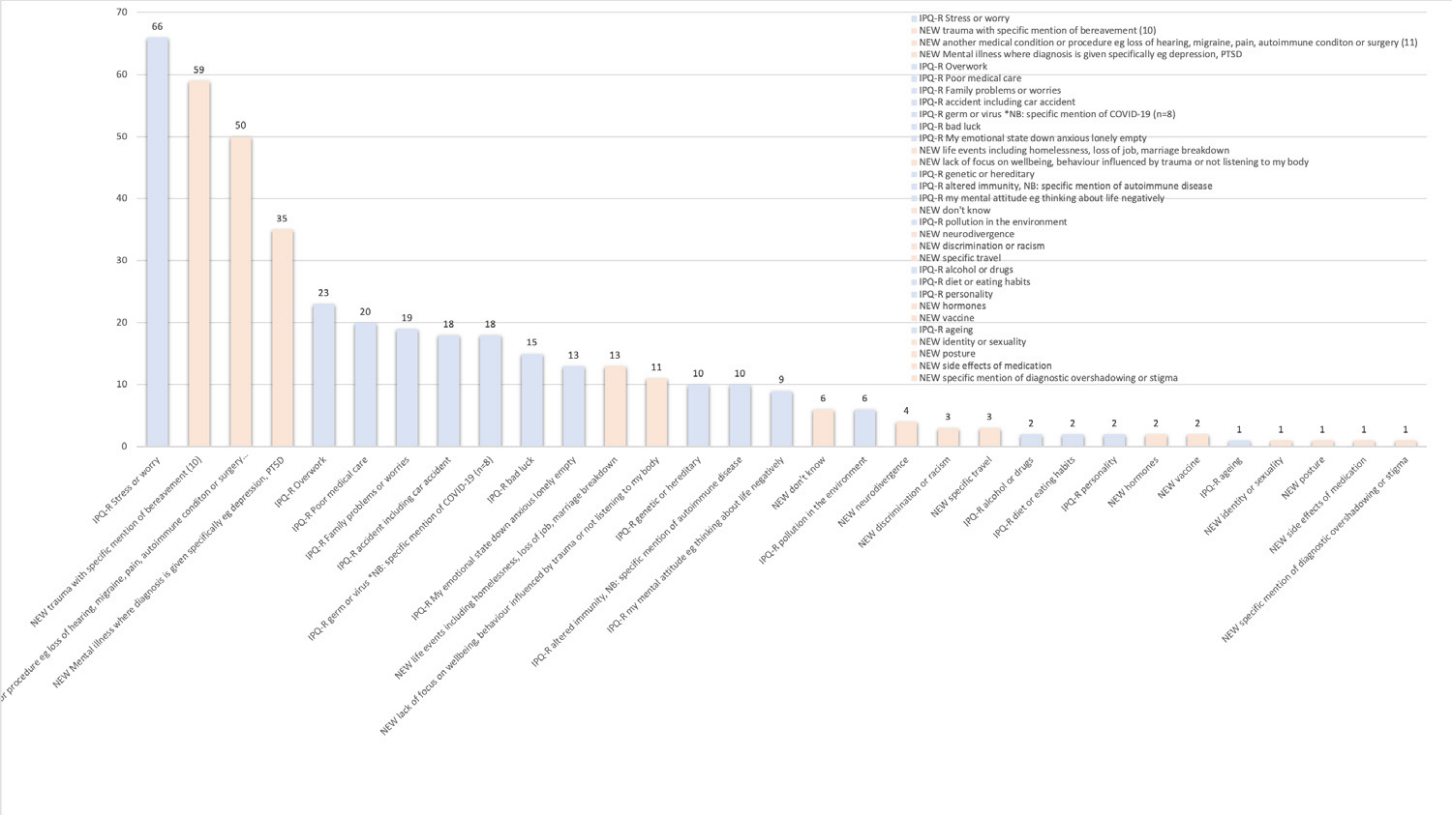
IPQ-R, Illness Perceptions Questionnaire Revised; PTSD, post-traumatic stress disorder.

Participants provided free-text responses for causes of their FND, which could include those already in the IPQ-R list or new causes, with instructions specifically requesting they enter what they believe, rather than what others including doctors have told them. The frequencies of these are described in figure 2. 58 (95.0%) of survey participants provided free-text responses at baseline, 44 (72.1%) immediately following the session and 40 (65.6%) at 1 month. The most common reported cause was stress (66 total responses), already included in the IPQ-R. New causes not currently included in IPQ-R included trauma (59 responses with 11 specifically mentioning childhood abuse and 10 traumatic bereavement). The third most common cause identified was another medical condition/procedure (50 responses, including 10 describing surgery, and 11 describing migraine). There were also 35 causal attributions of FND to a specific mental illness diagnosis, (eg, depression, post-traumatic stress disorder (PTSD)). IPQ-R already has a category for germ or virus, however eight participants specifically mentioned COVID-19.

Six participants identified a new cause following the education session which fit broadly under the headings of virus, emotional distress and trauma. 29 participants reordered their causes.

### Correlations

Lower social functioning (measured with SF-36) was associated with higher scores on SSCI-8 (0.52, 95% CI 0.31 to 0.67, p<0.001) and CIASS (0.45, 95% CI 0.22 to 0.64, p<0.001) in 59 participants at baseline. Higher scores of FND-associated distress, measured on the IPQ-R emotional representations subscale were correlated with lower scores of emotional well-being on SF-36 (0.60, 95% CI 0.40 to 0.74, p<0.001), and engagement (PHE) (0.76, 95% CI 0.61 to 0.85, p<0.001). Higher distress on IPQ-R was also associated with higher anxiety, measured by HADS (0.59, 95% CI 0.39 to 0.74, p<0.001) and higher enacted and internalised stigma on SSCI-8 (0.60, 95% CI 0.43 to 0.75, p<0.001). Higher levels of stigma on SSCI-8 (0.55, 95% CI 0.33 to 0.71, p<0.001) and anticipated stigma from colleagues were associated with increased scores on HADS depression scales (0.54, 95% CI 0.32 to 0.70, p<0.001). Higher levels of enacted and internalised stigma (SSCI-8) correlated with anticipated stigma (CIASS) (0.59, 95% CI 0.38 to 0.73, p<0.001).

### Questions and unmet needs

**Figure 3:**
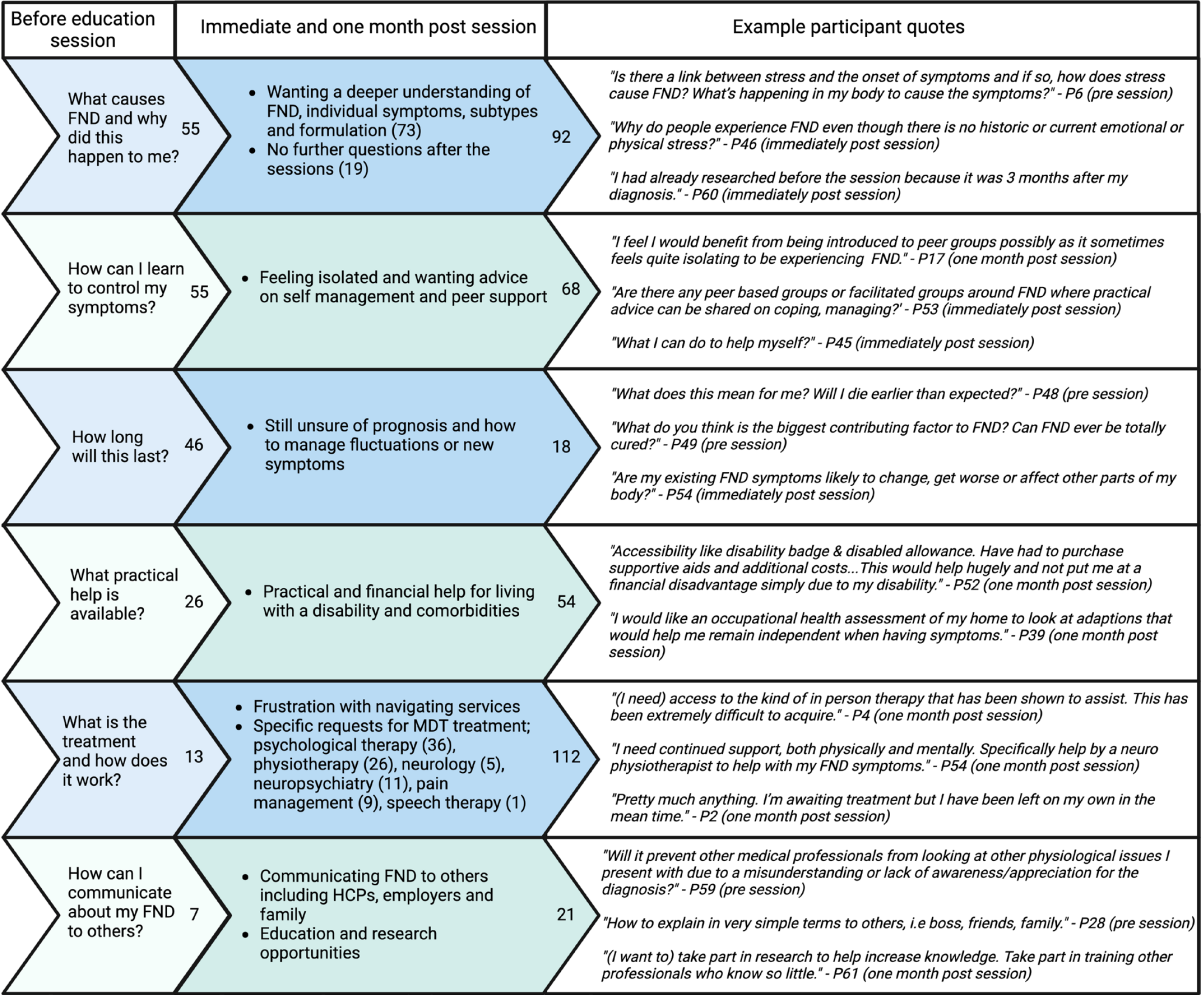
Content analysis of questions and unmet needs before, immediately and 1 month after the education session. The numbers on the right side of the arrows indicate the total number of responses within each category. FND, functional neurological disorder; HCP, healthcare professional; MDT. multidisciplinary team.

Content analysis of free-text responses and frequencies are described in figure 3. At baseline, the most common question or need related to what caused FND and why it affected the individual. Participants also wanted help with communicating about FND and sought advice on living with a disability.

As figure 3 demonstrates needs and questions evolved from more general to specific questions and requests following the education session, but remained similar immediately after and 1 month after the session, so these timepoints were analysed together. After the session there were requests for individual formulation and FND subtype information, practical and peer support, help with navigating services and treatments, including precise identification of need for MDT and interest in research. Quotes such as the one from P17 highlight the isolation of living with FND, while P48 expresses prognostic uncertainty.

Many expressed frustration with difficulties accessing treatment despite knowing what they needed, as exemplified in quotes from P4 and P2. Participants identified concerns about lack of FND knowledge in healthcare professionals (eg, P61), and fear of diagnostic overshadowing (eg, P59). There were 11 and 9 instances, respectively of no further questions immediately following and 1 month after the session, for example, “*The session was altogether very informative and answered many questions I had not thought to ask and addressed many of my concerns”*—P58.

## Discussion

This study examined illness perceptions, engagement and experiences of stigma in people with a variety of FND subtypes attending a single multidisciplinary education session. We found small but significant changes in illness coherence and engagement suggesting the session may have contributed to improved understanding about FND, and willingness to enter treatment. Although the study lacked a control group, free-text responses suggested after the session participants felt generally better informed and wished to learn more about their individual FND, and were seeking advice on self-management, access to multidisiplinary treatment and peer support.

### Comparison with other studies measuring illness perceptions

Our sample’s baseline illness perception scores were broadly similar to a previous study in both functional seizures (FS) and weakness (FW) across subscales measuring consequences, treatment control and emotional distress.^13^ Although direct comparison is difficult, our sample appeared to have higher levels of belief in personal control (67% mean 20.03 (our sample) vs 42% FS and 46% FW) and coherence (54% mean 13.03 (our sample) vs 32% in FS and 24% in FW) even at baseline. This may reflect increasing online FND information provision (eg, neurosymptoms.org and charities such as FND Hope, high-quality accessible webinars from the Functional Neurological Disorder Society), and increasing positive diagnosis^8^ compared with 2015 when the comparison paper was published. This growing online resource availability may also have implications for the education session impact, where attendees who have already researched their condition already have a high baseline theoretical knowledge. A few comments including from P3 support this “*I had already researched before the session because it was 3 months after my diagnosis*”.

Our sample wanted more personalised information about their FND as well as access to peer support. This indicates a potential role for a general FND session and then smaller group interventions focused on subtypes. For example a three session CBT-informed education group for functional seizures found improvement in frequency of attacks and illness beliefs related to prognosis and understanding.^33^ Guided self-help CBT educational workbook interventions where patients also access a few meetings with a therapist^34^ have been also associated with global improvement. This would suggest that personalisation of treatment, either through attending a group for a specific FND subtype or having the opportunity to discuss symptoms and formulation with a professional, is valuable.

While we did not find any change in subscales measuring hope about treatment efficacy or prognosis, evidence shows changes in these beliefs can be achieved through intensive treatment. A 4-week multidisciplinary FND treatment programme found significant improvements in perceptions related to chronicity, coherence, emotional representations and consequence.^31^

### Relationships between functioning, quality of life, illness perceptions and stigma

Our sample reported significant functional impairment, poor quality of life and high levels of distress associated with their FND. Higher levels of stigma were associated with lower social functioning. Distress associated with FND was also linked with poorer emotional well-being and lower engagement. This supports the findings of a recent systematic review of illness representations in both functional and epileptic seizures, which found that threatening perceptions were associated with higher distress.^11^ Our findings also suggest the emotional and functional impact of FND is not solely related to symptoms themselves, but is also linked to the response of a person’s community, employers, family and healthcare professionals, although the direction of effect cannot be ascertained from this study. Kirmayer and Gomez-Carillo expanding on bio-psycho-social formulations in functional conditions, suggest a concept of looping where the experience of a condition and sense of agency is constantly reconfigured through interactions with physical and social experiences, including access to power and resources.^35^

### Occupational and financial impacts of FND

Higher anticipated stigma from employers was linked to increased depressive scores on HADS. 51.7% of our sample reported serious financial consequences resulting from FND. Qualitative responses described needs for practical information on living with a disability and help in communicating about FND, including to employers. A global survey conducted by FND Hope found that 88% of participants reported work difficulties including performance issues, accidents or symptom exacerbation.^36^ There is increasing awareness of the need for occupational therapy within multidisciplinary management of FND.^8 37^ Our findings and existing literature suggest people with FND need advice on negotiating reasonable adjustments to function optimally and mitigate financial effects of their condition, including accessing benefits if appropriate.^36^

### Factors contributing to stigma in FND

Our participant cohort experienced higher enacted and internalised stigma (SSCI-8) scores (mean 20 (95% CI 18.48 to 23.00)) compared with a group of people with Multiple Sclerosis (MS) (mean 12.23) and Parkinson’s disease (mean 12.07).^26^ Experienced stigma (SSCI-8) was correlated with anticipated stigma (CIASS), suggesting past experiences may contribute to future expectations. Anticipated stigma is linked to poorer quality of life in epilepsy, fibromyalgia and lupus, but can be mediated by social support.^38^ In our sample, higher stigma scores (SSCI-8 and CIASS) were associated with lower self-reported social functioning (SF-36). It has been suggested that experiences of stigma in FND lead to social withdrawal, reduced disclosure and self-blame.^19^ A recent review^39^ highlighted how people with functional seizures may be prone to shame and associated comorbidities such as PTSD, and experience shame-inducing and stigmatising interactions,^40^ which may represent a link between predisposing and perpetuating factors. Free-text responses in our study echoed these experiences, and described desire for peer connection.

The high levels of stigma participants anticipated from healthcare professionals is unsurprising given the long duration of symptoms, lack of knowledge and negative attitudes from professionals^15^ and discrimination identified in previous studies.^8^ A service evaluation of health and social care experiences found compared with people with MS, those with FND were more likely to feel misunderstood and not treated with dignity by their doctors, and had more difficulties accessing diagnosis, specialists and coordinated care.^41^ A recent synthesis of stigma in FND formulated that invalidating clinical interactions contribute to hesitancy in seeking help and negative self-evaluations.^19^

### The relationship between hope, chronicity and access to treatment

The recently published Optimal Care Pathway from the UK National Neurological Advisory Group, which was a collaborative development exercise between professionals and experts by experience, identified numerous barriers to care for people with FND, including accessing community therapies and frequent discharge from services without treatment.^8^ Participants in our study described a lack of knowledge of FND in healthcare professionals encountered outside tertiary services, echoing the qualitative experiences of people with FND accessing non-specialist physiotherapy who described sometimes receiving inappropriate treatments and resulting harm.^42^ Our sample had a long duration of illness; 72.2% having had FND for more than a year. These factors may in part explain why they had low treatment hopefulness and a slightly increased anticipation of FND being a chronic condition following the education session.

A systematic review of modifiable factors of illness perceptions in chronic somatic conditions suggested psychosocial factors, information provision, satisfaction with information and quality of care are important to consider when tailoring education programmes.^43^ Although not specifically focused on FND, these findings in combination with correlations in our study suggest perceptions are not a discrete function of an illness itself and are instead in dynamic interaction with experiences (both positive and negative) and personal and healthcare relationships.

### Future directions

Given the relationship between illness perceptions, quality of life and engagement, our study supports the need for further work in developing an FND-specific adaptation of standard IPQ-R as suggested by its authors.^25^ In other conditions such as atrial fibrillation adaptation has been achieved through the use of qualitative ‘think aloud’ methodology and factor analysis.^25^ Such adaptation could elicit more detailed and nuanced experiences of people with FND. For example, trauma which is not currently included in the list of standard causes in IPQ-R, was identified by participants as the second most common self-reported cause, after stress.

However, our survey responses were often brief and the nature and timing of the trauma was not described. The relationship between trauma and FND is complex; from historical, outdated models of conversion disorder and more recent removal of the need to identify trauma to ‘explain’ the illness in the latest iteration of DSM.^44^ Contemporary studies have identified higher rates of childhood maltreatment in people with FND compared with controls, but also identified a group of people for whom this is not relevant.^44^ More refined understandings are emerging, with implications for treatment. A recent study explored differences between patients with or without comorbid PTSD and argued for a possible trauma FND subtype.^45^

Participants in our study identified a link between stress and their FND as well as other mental health conditions. This may also explain the frequent requests for psychological therapy. Larger sample sizes are needed before factor analyses or new ‘cause’ items could be added to an FND-specific IPQ-R. However, our findings support those of Butler *et al* surveying >1000 people with FND, where most participants attributed the cause of their FND to both physical and stress/trauma-related factors.^22^ Taken together this strongly suggests an expanded selection of causes should be explored, including consideration of medical conditions and surgeries as triggers for FND which is increasingly recognised.^46^ The variety of causes and needs identified by participants and comorbid conditions in the group also highlights the role of personalised formulations and support for navigating health systems with multimorbidity.

### Limitations

The survey response rate was relatively low (37%), with significant dropouts. However, this was not unexpected in a multiple time-point study with participants experiencing significant functional impairment and fatigue. The small sample size and lack of control group mean any association cannot be solely attributed to the education session. We did not repeat measures of mood, symptom burden or quality of life after the session, so relationships between those factors and illness perceptions or stigma could only be measured at baseline. Those attending an education session, and completing a survey are likely to be the most engaged and in agreement with their diagnosis. Additional studies are needed to gather the views of those who decline to attend an education session, are rendered unable to engage due to other comorbidities or face barriers within local services. The survey sample was largely white, and the survey and education sessions were provided in English only.

Our sample size may have been insufficient to detect improvements in beliefs about treatability found in the earlier evaluation.^21^ Recruitment for our study may have been more challenging due the sessions being online and greater questionnaire burden. Moving online may also have influenced the frequent requests for connection to others with FND, which would have been an inherent part of the prepandemic face-to-face session.

Although several of those conducting the education session were involved in writing this paper, the primary analysis was conducted by CB who had no role in the session itself, minimising bias in the analysis of results.

## Conclusions

This study found a single CBT-informed MDT education session was associated with increased understanding of FND and engagement. The cohort reported significant functional impairment, comorbidities, long duration of symptoms and high levels of stigma. Illness perceptions in FND should be conceptualised as being in dynamic relation with experiences of stigma, social and occupational functioning and challenges in accessing services. People with FND in our study expressed desire for personalised formulation explaining their individual subtype, peer support, self-management strategies and practical advice on navigating life with a disability.

Although a small sample, free-text causes reported by participants suggest that stress and trauma remain important considerations within individual formulations. The interaction of FND with comorbid medical conditions, surgeries and mental health diagnoses warrants further exploration. Future research should adapt the IPQ-R for FND, using qualitative and quantitative methods. Collaboration with people with lived experience of FND is vital to explore how experiences of stigma, accessing treatment and disability support affect internal representations. As so clearly identified by participants in our study—education is not only needed for those with FND, but also for healthcare professionals, employers, family and wider society. Education for individuals is only a gateway to effective management; patients with FND also need access to timely and appropriate treatment to improve symptoms and quality of life.

## Data Availability

All data relevant to the study are included in the article or uploaded as supplementary information.
