# Supplementary material for: Illness perceptions, experiences of stigma and engagement in functional neurological disorder (FND): exploring the role of multidisciplinary group education sessions
Source: BMJ Neurol Open. 2024 Jun 5;6(1):e000633. doi: 10.1136/bmjno-2024-000633 (PMC11163674; doi:10.1136/bmjno-2024-000633)
Supplement: Supplementary data [file bmjno-2024-000633supp001.pdf]

Tools Saved at 17:45 Draft Preview Publish

Experiences of FND: Pre-Education Session Survey

Section 1

Intro

Experiences, understanding and conceptual models in patients with functional neurological disorders (FND): a qua quantitative study

'Experiences of FND': Pre-Education Session Survey (Research Ethics Project ID: 268233)

Section 1 of 8.

Thank you for agreeing to take part in this survey. The survey has 8 sections, which will each take between 5 - 15 minutes. through at your own pace. If you need to take a break please leave the window on your computer open so you can continu needing to start again.

The whole survey will take no more than an hour.

The first section asks about some basic information and symptoms for which you are attending the St George's Functional N Disorders Education Session.

You will be asked to first enter your study ID. This is a random 6 digit number assigned to you in the email inviting you to th will need this number for this survey and to enter to subsequent surveys. If you are unable to locate your number, please e catherine.bailey@swlstg.nhs.uk and we will email it to you.

Some of the questions in the survey will ask about your mood and experiences of stigma related to your FND. We recognis these questions may cause distress. If you feel overwhelmed or distressed and need mental health support the details for c below and at the end of the survey:

If you live in any of the five of our London boroughs: Kingston, Merton, Richmond, Sutton and Wandsworth and you need n support in a crisis you can call the 24/7 Mental Health Crisis Line on 0800 028 8000.

If you are living outside of these areas you can visit this website and follow the steps for how to get help: https://www.nhs.u services/mental-health-services/where-to-get-urgent-help-for-mental-health/

Q1

Q1.
Please enter your 6 digit number from the email inviting you to the survey.

Q2

Q2.
Please enter the date of the FND education session you are attending (dd/mm/yyyy):

Q3

Q3.
Please enter your age in years:

Q4

Q4.
Please select the answer which best describes your ethnic background

- Asian Indian
- Asian Pakistani
- Asian Bangladeshi
- Asian Chinese
- Any other Asian background
- White-Asian
- White British
- White Irish
- White Gypsy or Irish Traveller
- Any other white background
- Black African
- Black Caribbean
- White and Black African
- White and Black Caribbean
- Any other Black background
- Arab
- Any other ethnic group

Q5

Q5.
Please select the option which best describes your gender:

- Man
- Woman
- Transgender man
- Transgender woman
- Non-binary
- Prefer not to say

Q6

Q6.
Please select the option which reflects your highest educational qualitifcation:

- Primary School
- GCSEs or equivalent
- A-levels or equivalent
- University undergraduate degree
- University postgraduate degree
- Doctoral degree

Q7

Q7.
Please select the option which best describes your current relationship status:

- ○ Married/civil partner
- ○ Relationship but not married or civil partnered
- ○ Single
- ○ Widowed
- ○ Divorced or separated

Q8

Q8.
Please select the option which best describes your current employment status:

- ○ Full time work
- ○ Part time work
- ○ Retired (due to age)
- ○ Retired (due to medical condition)
- ○ Volunteer work
- ○ Short term sick leave (<3 months)
- ○ Long term sick leave (>3 months)
- ○ Student

Q9

Q9.
Are you in receipt of any benefits?

- ○ No
- ○ Yes

Q10

Q10.
With regards to your functional neurological disorder (FND) please select the primary (or main) symptom for which you are
FND Education Session:

- ○ Movement disorder (uncontrollable movements)
- ○ Gait disturbance (trouble walking)
- ○ Tremor (shaking)
- ○ Weakness or paralysis
- ○ Muscle dystonia (stiffness or spasms)
- ○ Seizures or fits
- ○ Memory or thinking problems
- ○ Numbness or tingling
- ○ Other

Q11

Q11.
Please estimate how long you have experienced the above symptom:

- ◯ Less than 6 weeks
- ◯ 6 weeks - 3 months
- ◯ 3 months - 6 months
- ◯ 6 months - 12 months
- ◯ 1 - 5 years
- ◯ More than 5 years

Q12

Q12.
Please list any names or diagnoses healthcare professionals have given to describe your symptom (put none or nil if you ar
of having been given a diagnosis):

Q13

Q13.
In addition to your primary or main symptom (described in question 11) please also select any other symptoms you also ex
Select as many as apply to you.

- ☐ Movement disorder (uncontrollable movements)
- ☐ Gait disturbance (trouble walking)
- ☐ Tremor (shaking)
- ☐ Weakness or paralysis
- ☐ Seizures or fits
- ☐ Memory or thinking problems
- ☐ Numbness or tingling
- ☐ Headache
- ☐ Body pain
- ☐ Loss of balance
- ☐ Bladder problems
- ☐ Bowel problems
- ☐ Fatigue
- ☐ Difficulty talking
- ☐ Difficulty swallowing
- ☐ Visual problems
- ☐ Low mood or depression
- ☐ Anxiety
- ☐ Panic attacks
- ☐ Post traumatic stress disorder (PTSD)
- ☐ Other mental health problem
- ☐ Other

Bailey C, *et al. BMJ Neurol Open* 2024; 6:e000633. doi: 10.1136/bmjno-2024-000633

Q14

Q14.
Thinking ahead to the FND education session, what question or questions would you most like answered about your FND?

Import from library

Section 2

Section 2 of 8.
The following questions are taken from the RAND SF36 Item Survey (v1.0) and will ask about your health generally. This se
around 10 minutes.

Q1

Q1. In general, would you say your health is:

- Excellent
- Very Good
- Good
- Fair
- Poor

Q2

Q2. Compared to one year ago, how would you rate your health in general now?

- Much better now than one year ago
- Somewhat better now than one year ago
- About the same
- Somewhat worse now than one year ago
- Much worse now than one year ago

Bailey C, *et al. BMJ Neurol Open* 2024; 6:e000633. doi: 10.1136/bmjno-2024-000633

Q3-12

Q3 - 12. The following items are about activities you might do during a typical day. Does your health now limit you in these so, how much? (Please tick the box which best describes your experiences)

	Yes, limited a lot	Yes, limited a little	No, not lim
3. Vigorous activities, such as running, lifting heavy objects, participating in strenuous sports	○	○	○
4. Moderate activities, such as moving a table, pushing a vacuum cleaner, bowling or playing golf	○	○	○
5. Lifting or carrying groceries	○	○	○
6. Climbing several flights of stairs	○	○	○
7. Climbing one flight of stairs	○	○	○
	Yes, limited a lot	Yes, limited a little	No, not lim
8. Bending, kneeling or stooping	○	○	○
9. Walking more than one mile	○	○	○
10. Walking several blocks	○	○	○
11. Walking one block	○	○	○
12. Bathing or dressing yourself	○	○	○

Q4

Q13 - 16. During the past 4 weeks, have you had any of the following problems with your work or other regular daily activiti of your physical health?

	Yes	No
13. Cut down the amount of time you spent on work or other activities	○	○
14. Accomplished less than you would like	○	○
15. Were limited in the kind of work or other activities	○	○
16. Had difficulty performing the work or other activities (for example, it took extra effort)	○	○

Q5

Q17 - 19. During the past 4 weeks, have you had any of the following problems with your work or other regular daily activiti of any emotional problems (such as feeling depressed or anxious)?

	Yes	No
17. Cut down the amount of time you spent on work or other activities	○	○
18. Accomplished less than you would like	○	○
19. Didn't do work or other activities as carefully as usual	○	○

Q20. During the past 4 weeks, to what extent has your physical health or emotional problems interfered with your normal s with family, friends, neighbours, or groups?

- ○ Not at all
- ○ Slightly
- ○ Moderately
- ○ Quite a bit
- ○ Extremely

Q7

Q21. How much bodily pain have you had during the last 4 weeks?

- None
- Very mild
- Mild
- Moderate
- Severe
- Very severe

Q8

Q22. During the past 4 weeks, how much did pain interfere with your normal work (including both work outside the home a housework)?

- Not at all
- Slightly
- Moderately
- Quite a bit
- Extremely

Q71

Q23. How much of the time during the past 4 weeks...did you feel full of pep?

- All of the time
- Most of the time
- A good bit of the time
- Some of the time
- A little of the time
- None of the time

Q72

Q24. How much of the time during the past 4 weeks...have you been a very nervous person?

- All of the time
- Most of the time
- A good bit of the time
- Some of the time
- A little of the time
- None of the time

Q73

Q25. How much of the time during the past 4 weeks... Have you felt so down in the dumps that nothing could cheer you up

- All of the time
- Most of the time
- A good bit of the time
- Some of the time
- A little of the time
- None of the time

Q74

Q26. How much of the time during the past 4 weeks... Have you felt calm and peaceful?

- All of the time
- Most of the time
- A good bit of the time
- Some of the time
- A little of the time
- None of the time

Q75

Q27. How much of the time during the past 4 weeks... Did you have a lot of energy?

- All of the time
- Most of the time
- A good bit of the time
- Some of the time
- A little of the time
- None of the time

Q76

Q28. How much of the time during the past 4 weeks... Have you felt downhearted and blue?

- All of the time
- Most of the time
- A good bit of the time
- Some of the time
- A little of the time
- None of the time

Q77

Q29. How much of the time during the past 4 weeks... Did you feel worn out?

- All of the time
- Most of the time
- A good bit of the time
- Some of the time
- A little of the time
- None of the time

Q78

Q30. How much of the time during the past 4 weeks... Have you been a happy person?

- All of the time
- Most of the time
- A good bit of the time
- Some of the time
- A little of the time
- None of the time

Q79

Q31. How much of the time during the past 4 weeks... Did you last feel tired?

- All of the time
- Most of the time
- A good bit of the time
- Some of the time
- A little of the time
- None of the time

Q10

Q32. During the past 4 weeks, how much of the time has your physical health or emotional problems interfered with your s[illegible]
(like visiting with friends, relatives, etc.)?

- All of the time
- Most of the time
- Some of the time
- A little of the time
- None of the time

Q80

Q33. How TRUE or FALSE is the following statement for you:
I seem to get sick a little easier than other people

- Definitely true
- Mostly true
- Don't know
- Mostly false
- Definitely false

Q81

Q34. How TRUE or FALSE is the following statement for you:
I am as healthy as anybody I know

- Definitely true
- Mostly true
- Don't know
- Mostly false
- Definitely false

Q82

Q35. How TRUE or FALSE is the following statement for you:
I expect my health to get worse

- Definitely true
- Mostly true
- Don't know
- Mostly false
- Definitely false

Q83

Q36. How TRUE or FALSE is the following statement for you:
My health is excellent

- Definitely true
- Mostly true
- Don't know
- Mostly false
- Definitely false

Import from library

Section 3

EQ5D5L

Section 3 of 8.

The following questions are taken from the EQ5D5L survey, which asks about how you manage with day to day tasks. Thes
will take around 5 minutes to complete.

Health Questionnaire English version for the UK © EuroQol Research Foundation. EQ-5D™ is a trade mark of the EuroQol F
Foundation. UK (English) v2.1

Q1

Q1. Please select the ONE option which best describes your health TODAY.

MOBILITY:

- ○ I have no problems in walking about
- ○ I have slight problems in walking about
- ○ I have moderate problems in walking about
- ○ I have severe problems in walking about
- ○ I am unable to walk about

Q2

Q2. Please select the ONE option which best describes your health TODAY.

SELF-CARE:

- ○ I have no problems washing or dressing myself
- ○ I have slight problems washing or dressing myself
- ○ I have moderate problems washing or dressing myself
- ○ I have severe problems washing or dressing myself
- ○ I am unable to wash or dress myself

Q3

Q3. Please select the ONE option which best describes your health TODAY.

USUAL ACTIVITIES (e.g. work, study, housework, family or leisure activities):

- ○ I have no problems doing my usual activities
- ○ I have slight problems doing my usual activities
- ○ I have moderate problems doing my usual activities
- ○ I have severe problems doing my usual activities
- ○ I am unable to do my usual activities

Q4

Q4. Please select the ONE option which best describes your health TODAY.

PAIN/DISCOMFORT:

- ○ I have no pain or discomfort
- ○ I have slight pain or discomfort
- ○ I have moderate pain or discomfort
- ○ I have severe pain or discomfort
- ○ I have extreme pain or discomfort

Q5

Q5. Please select the ONE option which best describes your health TODAY.

ANXIETY/DEPRESSION:

- ◯ I am not anxious or depressed
- ◯ I am slightly anxious or depressed
- ◯ I am moderately anxious or depressed
- ◯ I am severely anxious or depressed
- ◯ I am extremely anxious or depressed

Q6

Q6. We would like to know how good or bad your health is **TODAY**.

You will see a scale numbered from 0 to 100.
100 means the **best** health you can imagine.
0 means the **worst** health you can imagine.

	0	10	20	30	40	50	60	70	80	90
Please indicate on the scale how your health is TODAY.										

Import from library

Section 4

IPQ-R

Section 4 of 8.

The following questions are taken from the Illness Perception Questionnaire - Revised (IPQ-R). This is a survey which has b
wide range of illnesses but has not yet been specifically adapted for FND. Your responses will be helpful in understanding h
survey can be used in FND. If any questions cause distress or do not seem to apply in FND, we would be grateful if you wo
answer them, but you can also leave any comments in a free text box at the end of this section.

This section will take around 10 minutes to complete.

IPQ-R

Listed below are a number of symptoms that you may or may not have experienced since your functional neurological diso
Please indicate by selecting Yes or No, whether you have experienced any of these symptoms since your illness (your FND
whether you believe that these symptoms are related to your FND.

Q1

Q1. I have experienced this symptom *since my FND*.

	Yes	No
Pain	○	○
Sore throat	○	○
Nausea	○	○
Breathlessness	○	○
Weight loss	○	○
	Yes	No
Fatigue	○	○
Stiff joints	○	○
Sore eyes	○	○
Wheeziness	○	○
Headaches	○	○
	Yes	No
Upset stomach	○	○
Sleep difficulties	○	○
Loss of strength	○	○

Q2

Q2. This symptom is *related to my FND*.

	Yes	No
Pain	○	○
Sore throat	○	○
Nausea	○	○
Breathlessness	○	○
Weight loss	○	○
	Yes	No
Fatigue	○	○
Stiff joints	○	○
Sore eyes	○	○
Wheeziness	○	○
Headaches	○	○
	Yes	No
Upset stomach	○	○
Sleep difficulties	○	○
Loss of strength	○	○

IP1

Views about your FND:

We are interested in your own personal views of how you now see your current illness (FND) or your primary symptom for w
have been referred for the education session. Please indicate how much you agree or disagree with the following statemen
FND by selecting the appropriate button.

3.
My FND will last a short time.

- Strongly disagree
- Disagree
- Neither agree nor disagree
- Agree
- Strongly agree

IP2

4.
My FND is likely to be permanent rather than temporary.

- Strongly disagree
- Disagree
- Neither agree nor disagree
- Agree
- Strongly agree

IP3

5.
My FND will last for a long time.

- Strongly disagree
- Disagree
- Neither agree nor disagree
- Agree
- Strongly agree

IP4

6.
My FND will pass quickly.

- Strongly disagree
- Disagree
- Neither agree nor disagree
- Agree
- Strongly agree

Bailey C, *et al. BMJ Neurol Open* 2024; 6:e000633. doi: 10.1136/bmjno-2024-000633

IP5

7.
I expect to have FND for the rest of my life.

- ◯ Strongly disagree
- ◯ Disagree
- ◯ Neither agree nor disagree
- ◯ Agree
- ◯ Strongly agree

IP6

8.
My FND is a serious condition.

- ◯ Strongly disagree
- ◯ Disagree
- ◯ Neither agree nor disagree
- ◯ Agree
- ◯ Strongly agree

IP7

9.
My FND has major consequences on my life.

- ◯ Strongly disagree
- ◯ Disagree
- ◯ Neither agree nor disagree
- ◯ Agree
- ◯ Strongly agree

IP8

10.
My FND does not have much effect on my life.

- ◯ Strongly disagree
- ◯ Disagree
- ◯ Neither agree nor disagree
- ◯ Agree
- ◯ Strongly agree

IP9

11.
My FND strongly affects the way others see me.

- ◯ Strongly disagree
- ◯ Disagree
- ◯ Neither agree nor disagree
- ◯ Agree
- ◯ Strongly agree

Bailey C, *et al. BMJ Neurol Open* 2024; 6:e000633. doi: 10.1136/bmjno-2024-000633

IP10

12.
My FND has serious financial consequences.

- Strongly disagree
- Disagree
- Neither agree nor disagree
- Agree
- Strongly agree

IP11

13.
My FND causes difficulties for those who are close to me.

- Strongly disagree
- Disagree
- Neither agree nor disagree
- Agree
- Strongly agree

IP12

14.
There is a lot which I can do to control my symptoms.

- Strongly disagree
- Disagree
- Neither agree nor disagree
- Agree
- Strongly agree

IP13

15.
What I do can determine whether my illness gets better or worse.

- Strongly disagree
- Disagree
- Neither agree nor disagree
- Agree
- Strongly agree

IP14

16.
The course of my FND depends on me.

- Strongly disagree
- Disagree
- Neither agree nor disagree
- Agree
- Strongly agree

IP15

17.
Nothing I do will affect my FND.

- Strongly disagree
- Disagree
- Neither agree nor disagree
- Agree
- Strongly agree

IP16

18.
I have the power to affect my FND.

- Strongly disagree
- Disagree
- Neither agree nor disagree
- Agree
- Strongly agree

IP17

19.
My actions will have no effect on the outcome of my FND.

- Strongly disagree
- Disagree
- Neither agree nor disagree
- Agree
- Strongly agree

Bailey C, *et al. BMJ Neurol Open* 2024; 6:e000633. doi: 10.1136/bmjno-2024-000633

IP18

20.
My FND will improve in time.

- Strongly disagree
- Disagree
- Neither agree nor disagree
- Agree
- Strongly agree

IP19

21.
There is very little that can be done to improve my FND.

- Strongly disagree
- Disagree
- Neither agree nor disagree
- Agree
- Strongly agree

IP20

22.
My treatment will be effective in curing my FND.

- Strongly disagree
- Disagree
- Neither agree nor disagree
- Agree
- Strongly agree

IP21

23.
The negative effects of my FND can be prevented (avoided) by my treatment.

- Strongly disagree
- Disagree
- Neither agree nor disagree
- Agree
- Strongly agree

IP22

24.
My treatment can control my FND.

- Strongly disagree
- Disagree
- Neither agree nor disagree
- Agree
- Strongly agree

IP23

25.
There is nothing which can help my condition (FND).

- ◯ Strongly disagree
- ◯ Disagree
- ◯ Neither agree nor disagree
- ◯ Agree
- ◯ Strongly agree

IP24

26.
The symptoms of my FND are puzzling to me.

- ◯ Strongly disagree
- ◯ Disagree
- ◯ Neither agree nor disagree
- ◯ Agree
- ◯ Strongly agree

IP25

27.
My FND is a mystery to me.

- ◯ Strongly disagree
- ◯ Disagree
- ◯ Neither agree nor disagree
- ◯ Agree
- ◯ Strongly agree

IP26

28.
I don't understand my FND.

- ◯ Strongly disagree
- ◯ Disagree
- ◯ Neither agree nor disagree
- ◯ Agree
- ◯ Strongly agree

IP27

29.
My FND doesn't make any sense to me.

- Strongly disagree
- Disagree
- Neither agree nor disagree
- Agree
- Strongly agree

IP28

30.
I have a clear picture or understanding of my FND.

- Strongly disagree
- Disagree
- Neither agree nor disagree
- Agree
- Strongly agree

IP29

31.
The symptoms of my FND change a great deal from day to day.

- Strongly disagree
- Disagree
- Neither agree nor disagree
- Agree
- Strongly agree

IP30

32.
My symptoms come and go in cycles.

- Strongly disagree
- Disagree
- Neither agree nor disagree
- Agree
- Strongly agree

IP31

33.
My FND is very unpredictable.

- Strongly disagree
- Disagree
- Neither agree nor disagree
- Agree
- Strongly agree

IP32

34.
I go through cycles in which my FND gets better and worse.

- ◯ Strongly disagree
- ◯ Disagree
- ◯ Neither agree nor disagree
- ◯ Agree
- ◯ Strongly agree

IP33

35.
I get depressed when I think about my FND.

- ◯ Strongly disagree
- ◯ Disagree
- ◯ Neither agree nor disagree
- ◯ Agree
- ◯ Strongly agree

IP34

36.
When I think about my FND I get upset.

- ◯ Strongly disagree
- ◯ Disagree
- ◯ Neither agree nor disagree
- ◯ Agree
- ◯ Strongly agree

IP35

37.
My FND makes me feel angry.

- ◯ Strongly disagree
- ◯ Disagree
- ◯ Neither agree nor disagree
- ◯ Agree
- ◯ Strongly agree

IP36

38.
My FND does not worry me.

- Strongly disagree
- Disagree
- Neither agree nor disagree
- Agree
- Strongly agree

IP37

39.
Having this illness (FND) makes me feel anxious.

- Strongly disagree
- Disagree
- Neither agree nor disagree
- Agree
- Strongly agree

IP38

40.
My FND makes me feel afraid.

- Strongly disagree
- Disagree
- Neither agree nor disagree
- Agree
- Strongly agree

Q4

41.
Causes of your illness:
We are interested in what **you** consider may have been the cause of your illness. As people are very different, there is no c
for these questions. We are most interested in your own views about the factors that caused your illness rather than what o
including doctors or family may have suggested to you. Below is a list of possible causes for your illness. Please indicate h
agree or disagree that they were causes for you by ticking the appropriate box.

	Strongly disagree	Disagree	Neither agree nor disagree	Agree	St
Stress or worry	○	○	○	○	
Hereditary - it runs in my family	○	○	○	○	
A germ or virus	○	○	○	○	
Diet or eating habits	○	○	○	○	
Chance or bad luck	○	○	○	○	
Poor medical care in my past	○	○	○	○	
	Strongly disagree	Disagree	Neither agree nor disagree	Agree	St
Pollution in the environment	○	○	○	○	
My own behaviour	○	○	○	○	
My mental attitude, eg: thinking about life negatively	○	○	○	○	
Family problems or worries caused my illness	○	○	○	○	
Overwork	○	○	○	○	
My emotional state, eg: feeling down, lonely, anxious, empty	○	○	○	○	
	Strongly disagree	Disagree	Neither agree nor disagree	Agree	St
Ageing	○	○	○	○	
Alcohol	○	○	○	○	
Smoking	○	○	○	○	
Accident or injury	○	○	○	○	
My personality	○	○	○	○	
Altered immunity	○	○	○	○	
	Strongly disagree	Disagree	Neither agree nor disagree	Agree	St
Group					

Q5

42.
In the boxes below, please list in rank-order the three most important factors that you now believe caused YOUR illness. Yo
any of the items from the box above, or you may have additional ideas of your own.
The most important causes for me:

1 [ ]

2 [ ]

3 [ ]

Q138

43.
As we mentioned above the IPQ-R has not previously been validated or used much in FND, so some of the questions may s or not apply.

If you would like to, you can use this box to enter the numbers or details of any questions which caused offence, seemed ir you would like to comment on.

Import from library

Section 5 | 15 Questions

Section 6

SSCI-8

Section 6 of 8.

The following questions are taken from the SSCI-8 (Stigma Scale for Chronic Illness) and relate to your experience of FND. has been used in a number of other conditions but has also not been used in FND previously. We understand some of the c cause distress when you think about difficult experiences you may have had. Details on how to seek help if you feel overwh found at the end of the survey or in the study information.

This section will take around 5 minutes to complete.

+ Add page break

Q1

1.
Please select the option which best matches your experience.

	Never	Rarely	Sometimes	Often
Because of my FND, some people avoided me	○	○	○	○
Because of my FND, I felt left out of things	○	○	○	○
Because of my FND, people avoided looking at me	○	○	○	○
I feel embarrassed about my FND	○	○	○	○
	Never	Rarely	Sometimes	Often
Because of my FND, some people seemed uncomfortable with me	○	○	○	○
I felt embarrassed because of my physical limitations	○	○	○	○
Because of my FND people have been unkind to me	○	○	○	○
Some people acted as though it was my fault I had FND	○	○	○	○

Import from library

Section 7

CIASS

Section 7 of 8.

The following questions are taken from the Chronic Illness Anticipated Stigma Scale (CIASS). This part of the survey will tal minutes.

The statements listed below describe some of the ways that people who have other chronic illnesses are treated by others. has also not been used in FND previously and we recognise that thinking about some of these things may be distressing. W your answers will help us to better understand your experiences. Details on how to seek help if you are feeling overwhelme available at the end of the survey or if you need to stop the survey at any point you can find these details in the study infor provided.

Please read these statements and mark how likely you think that they could happen to you in the future.

Q1

1. First, think about how your friends and family members such as parents, sisters and brothers, and children will treat you How likely is it that they will treat you in the following ways because of your FND?

	Very unlikely	Unlikely	Somewhat likely	Likely
A friend or family member will be angry with you	○	○	○	○
A friend or family member will blame you for not getting better	○	○	○	○
A friend or family member will think that your illness is your fault	○	○	○	○
A friend or family member will not think as highly of you	○	○	○	○

Q2

2. Now, think about how your coworkers and employers will treat you in the future. If you are not currently employed, think coworkers and employers that you might have in the future. How likely is it that they will treat you in the following ways bec FND?

	Very unlikely	Unlikely	Somewhat likely	Likely
Your employer will not promote you	○	○	○	○
Someone at work will discriminate against you	○	○	○	○
Your employer will assign a challenging project to someone else	○	○	○	○
Someone at work will think you cannot fulfil your work responsibilities	○	○	○	○

Q3

3. Finally, think about how healthcare providers such as doctors, nurses, technicians, and secretaries who work at hospitals offices will treat you in the future. How likely is it that they will treat you in the following ways because of your FND?

	Very unlikely	Unlikely	Somewhat likely	Likely
A healthcare worker will be frustrated with you	○	○	○	○
A healthcare worker will give you poor care	○	○	○	○
A healthcare worker will blame you for not getting better	○	○	○	○
A healthcare worker will think you are a bad patient	○	○	○	○

Import from library

Section 8

PHE

Section 8 of 8.

The final questions are taken from the Patient Health Engagement questionnaire. Following, you will find 5 statements that a person might feel when thinking about their illness. Each sentence can be completed by indicating one of the 4 states or points between the states. Please indicate the state that best describes your feelings when you think about your FND.

EXAMPLE QUESTION

Following, you will find 5 statements that describe how a person might feel when about his/her disease. Each sentence can be completed by indicating one of the 4 stat intermediate points between two states. Please, indicate the state that better describe indicating the corresponding position.

Following an example to help you in answering the questions

Example 1
When I think about my health status...

I feel sad		I feel anxious		I feel resigned		I feel relaxed
●	○	○	○	○	○	○

if you were sad when thinking about you disease, you would indicate the first position

Example 2
When I think about my health status...

I feel sad		I feel anxious		I feel resigned		I feel relaxed
○	●	○	○	○	○	○

if you were in an intermediate position between sadness and anxiety when thinking about you disease, you v indicate the second position

Click to write the question text

Q1

1. Thinking about my FND

	I feel in blackout		I am in alarm		I am aware	
	○	○	○	○	○	○

Q2

2. Thinking about my FND

	I feel dazed		I am in trouble		I am conscious	
	○	○	○	○	○	○

Q3

3. Thinking about my FND

When I think about my illness I feel overwhelmed by emotions		I feel anxious every time a new symptom arises		I've gotten used to my illness/condition	
○	○	○	○	○	○

Q4

4. Thinking about my FND

I feel very discouraged due to my illness		I feel anxious when I try to manage my illness		I feel I've adjusted to my illness		I opt futu
○	○	○	○	○	○	

Q5

5. Thinking about my FND

I feel totally oppressed by my illness		I am upset when a new symptom arises		I feel I have accepted my illness		I car life
○	○	○	○	○	○	

End

Thank you so much for taking the time to complete this survey. You will receive a further briefer survey to complete after yc education session and 1 month later.

You will be contacted in the coming weeks with details on the voucher to thank you for your time.

If you have questions or wish to discuss any aspect of the study or need to access your anonymised study number please c catherine.bailey@swlstg.nhs.uk

If any aspects of the study have caused distress and you need to speak to a mental health professional urgently please finc crisis lines below or please dial 999 in case of emergency.

Crisis contacts:
If you live in any of the five of our London boroughs: Kingston, Merton, Richmond, Sutton and Wandsworth and you need n support in a crisis you can call the 24/7 Mental Health Crisis Line on 0800 028 8000.

If you are living outside of these areas you can visit this website and follow the steps for how to get help: https://www.nhs.u services/mental-health-services/where-to-get-urgent-help-for-mental-health/

Import from library

Add Block

End of Survey

We thank you for your time spent taking this survey.

Your response has been recorded.
