# Supplementary figures and images for: Illness perceptions, experiences of stigma and engagement in functional neurological disorder (FND): exploring the role of multidisciplinary group education sessions
Source: BMJ Neurol Open. 2024 Jun 5;6(1):e000633. doi: 10.1136/bmjno-2024-000633 (PMC11163674; doi:10.1136/bmjno-2024-000633)

SSCI-8

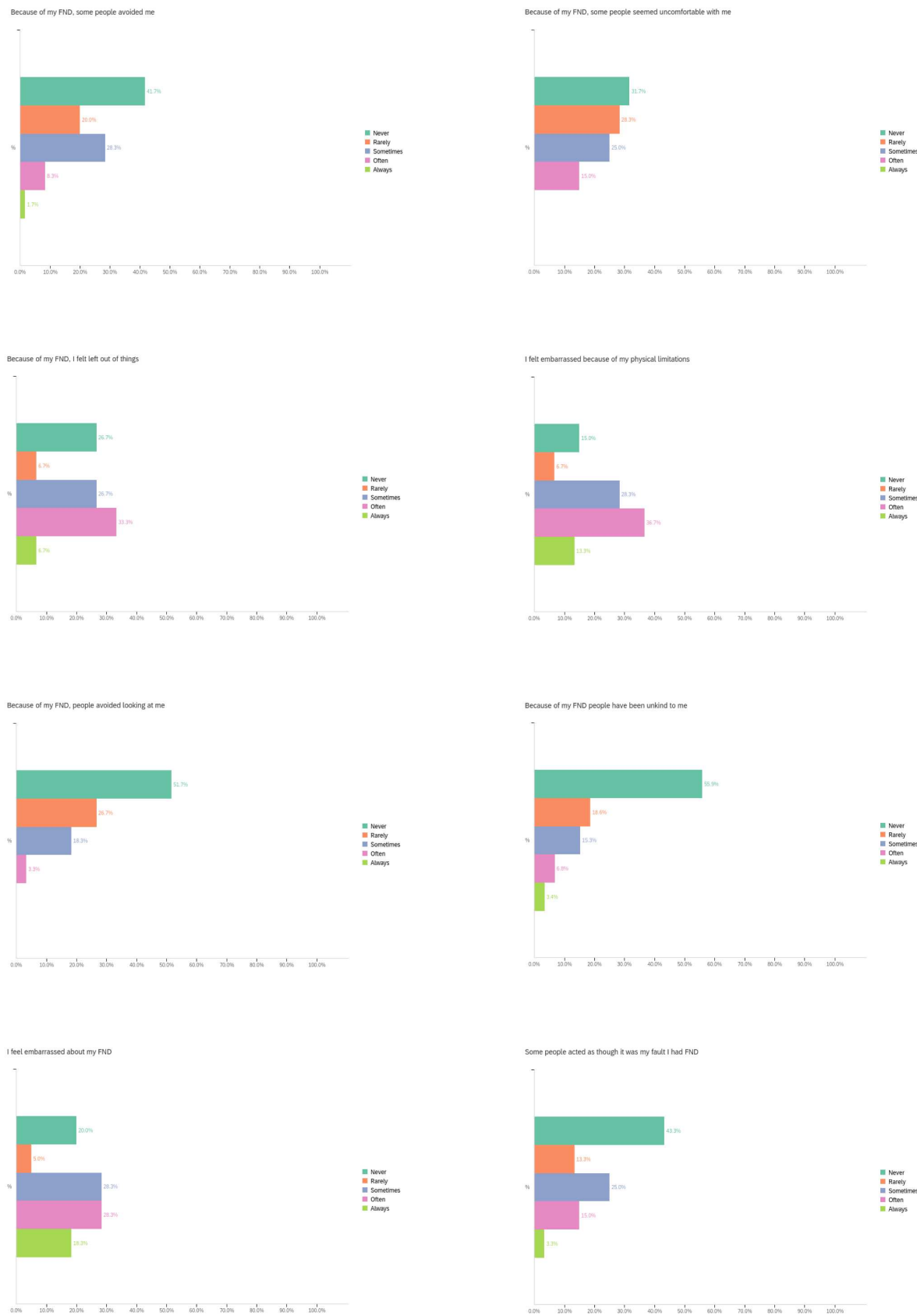

Supplement: Supplementary data [file bmjno-2024-000633supp002.pdf]
