# Supplementary material for: Illness perceptions, experiences of stigma and engagement in functional neurological disorder (FND): exploring the role of multidisciplinary group education sessions
Source: BMJ Neurol Open. 2024 Jun 5;6(1):e000633. doi: 10.1136/bmjno-2024-000633 (PMC11163674; doi:10.1136/bmjno-2024-000633)

CIASS – Friends and Family

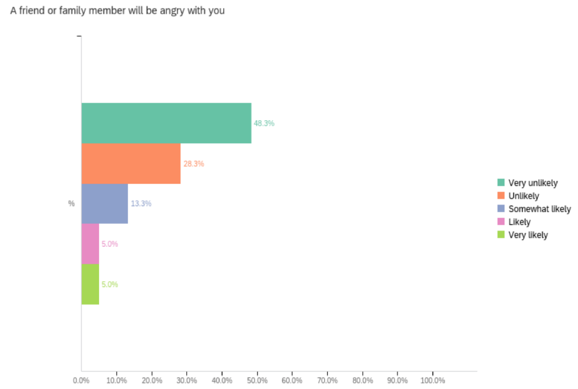

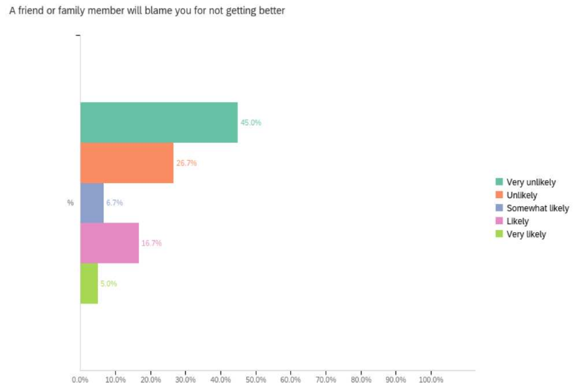

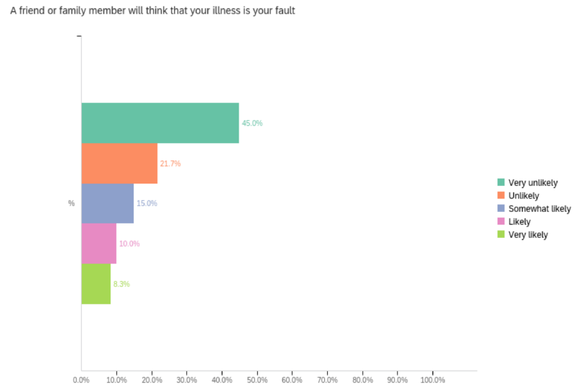

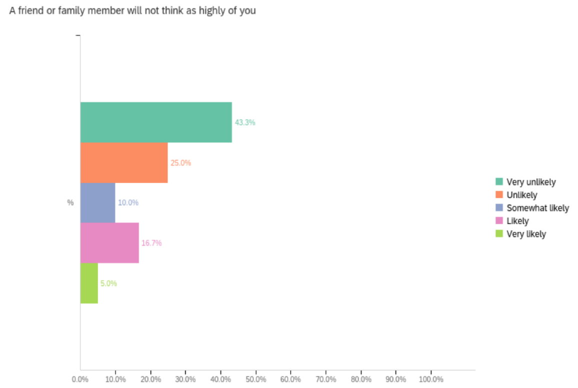

CIASS – Employers and Colleagues

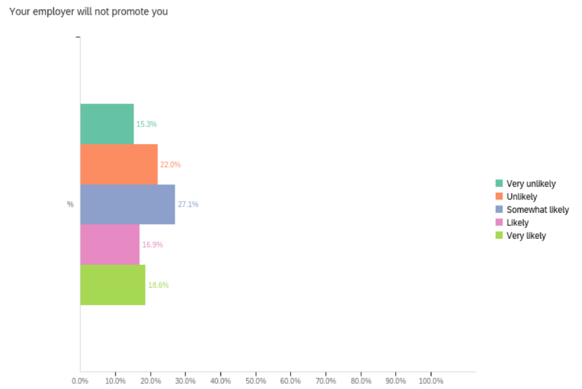

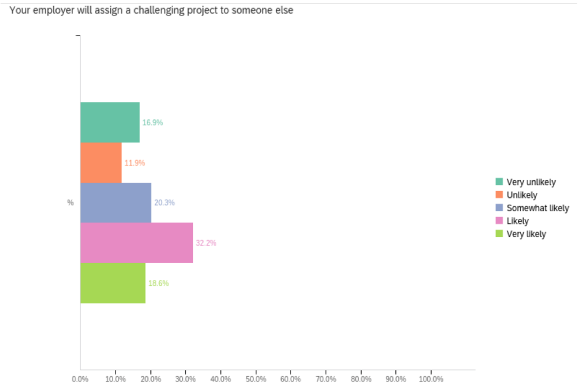

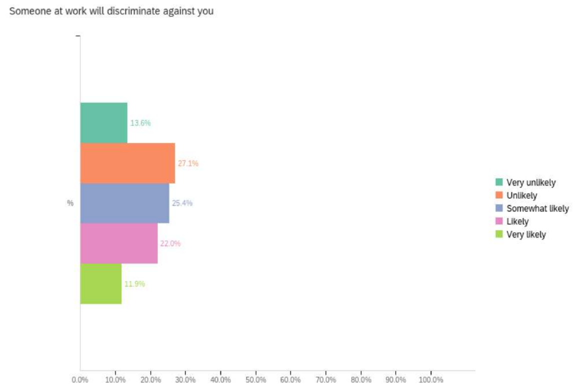

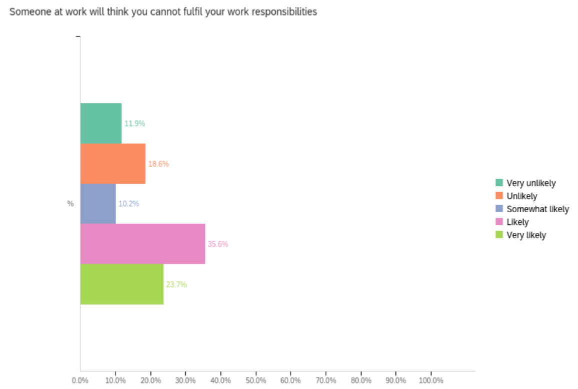

CIASS – Health Professionals

A healthcare worker will be frustrated with you

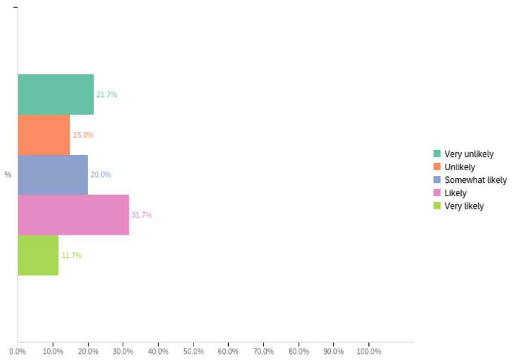

A healthcare worker will blame you for not getting better

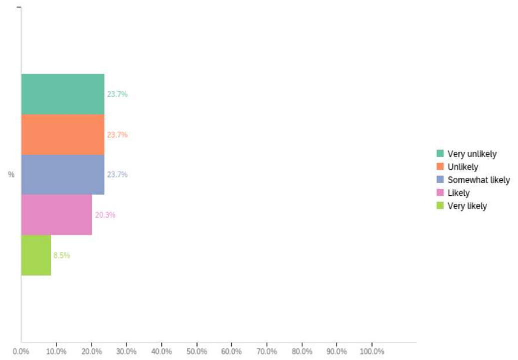

A healthcare worker will give you poor care

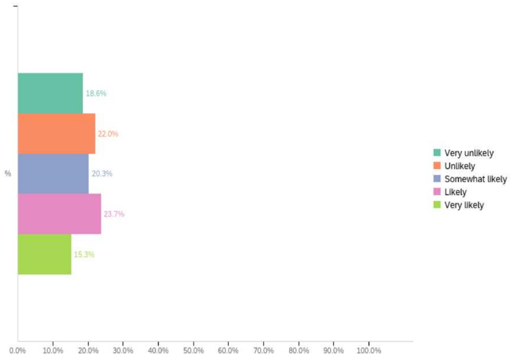

A healthcare worker will think you are a bad patient

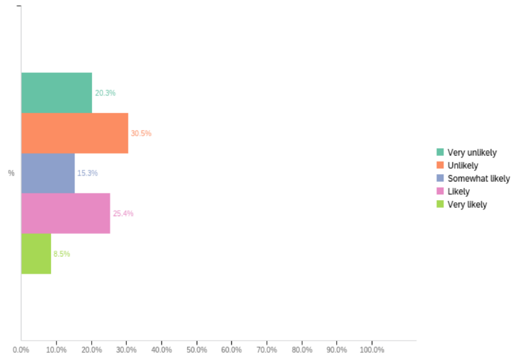

Supplement: Supplementary data [file bmjno-2024-000633supp003.pdf]
