# Supplementary material for: Illness perceptions, experiences of stigma and engagement in functional neurological disorder (FND): exploring the role of multidisciplinary group education sessions
Source: BMJ Neurol Open. 2024 Jun 5;6(1):e000633. doi: 10.1136/bmjno-2024-000633 (PMC11163674; doi:10.1136/bmjno-2024-000633)
Supplement: Supplementary data [file bmjno-2024-000633supp004.pdf]

IPQ-R Cause Baseline	Strongly disagree (%)	n	Disagree (%)	n	Neither agree nor disagree (%)	n	Agree (%)	n	Strongly agree (%)	n	Total (n)
Stress or worry	10.0	6	11.7	7	8.3	5	30.0	18	40.0	24	60
Hereditary - it runs in my family	46.7	28	25.0	15	21.7	13	5.0	3	1.7	1	60
A germ or virus	35.0	21	28.3	17	18.3	11	10.0	6	8.3	5	60
Diet or eating habits	28.3	17	36.7	22	26.7	16	8.3	5	0.0	0	60
Chance or bad luck	21.7	13	30.0	18	18.3	11	28.3	17	1.7	1	60
Poor medical care in my past	23.3	14	20.0	12	25.0	15	20.0	12	11.7	7	60
Pollution in the environment	43.3	26	36.7	22	18.3	11	0.0	0	1.7	1	60
My own behaviour	23.3	14	16.7	10	26.7	16	26.7	16	6.7	4	60
My mental attitude, eg: thinking about life negatively	21.7	13	20.0	12	26.7	16	26.7	16	5.0	3	60
Family problems or worries caused my illness	20.0	12	18.3	11	16.7	10	28.3	17	16.7	10	60
Overwork	23.3	14	18.3	11	11.7	7	26.7	16	20.0	12	60
My emotional state, eg: feeling down, lonely, anxious, empty	11.7	7	21.7	13	15.0	9	28.3	17	23.3	14	60
Ageing	43.3	26	35.0	21	11.7	7	10.0	6	0.0	0	60
Alcohol	58.3	35	23.3	14	13.3	8	3.3	2	1.7	1	60
Smoking	56.7	34	21.7	13	18.3	11	3.3	2	0.0	0	60
Accident or injury	35.0	21	10.0	6	21.7	13	16.7	10	16.7	10	60
My personality	31.7	19	28.3	17	15.0	9	16.7	10	8.3	5	60
Altered immunity	31.7	19	23.3	14	33.3	20	11.7	7	0.0	0	60
